# The Prognostic Values of the Insulin-Like Growth Factor Binding Protein Family in Ovarian Cancer

**DOI:** 10.1155/2020/7658782

**Published:** 2020-11-16

**Authors:** Ruoyi Zheng, Wenming Chen, Weiting Xia, Jingyu Zheng, Qing Zhou

**Affiliations:** ^1^Department of Gynecology, The First Affiliated Hospital of Wenzhou Medical University, Wenzhou, Zhejiang 325000, China; ^2^Department of Obstetrics, The First Affiliated Hospital of Wenzhou Medical University, Wenzhou, Zhejiang 325000, China; ^3^Department of Pathology, The First Affiliated Hospital of Wenzhou Medical University, Wenzhou, Zhejiang 325000, China

## Abstract

**Purpose:**

To assess the expression of insulin-like growth factor binding protein (IGFBP) family and its prognostic impact in ovarian cancer (OC) patients.

**Materials and Methods:**

The mRNA expression and protein expression of individual IGFBPs in healthy ovarian samples and OC tissues were explored through Oncomine, Gene Expression Profiling Interactive Analysis, and Human Protein Atlas database. Additionally, the prognostic values of the six IGFBP members in patients with OC were evaluated by Kaplan-Meier plotter.

**Results:**

IGFBP2 and IGFBP4 mRNA expression were remarkably upregulated in patients with OC. To be specific, the mRNA expression of IGFBP2 was upregulated in patients with serous ovarian cancer (SOC), while IGFBP1/3/4/5/6 mRNA levels were downregulated. In addition, the IGFBP4 protein expression was upregulated in SOC, and the IGFBP6 protein expression was upregulated in both of SOC and endometrioid ovarian cancer (EOC) tissues. High IGFBP1 mRNA levels showed favorable overall survival (OS) and progression-free survival (PFS) in all OC. Meanwhile, increased IGFBP5/6 mRNA levels revealed worsen OS and PFS in all OC patients. IGFBP4/6 mRNA levels predicted unfavorable OS and PFS only in SOC patients. Moreover, the aberrant mRNA expression of IGFBP1/2/4/5/6 was correlated with significantly prognosis in patients receiving different chemotherapeutic regimens.

**Conclusion:**

This study indicates that the IGFBP family reveals distinct prognosis in patients with OC. IGFBP1/2/4/5/6 are useful prognostic predictors for chemotherapeutic effect in OC patients, and IGFBP2/4 are potential tumor markers for the diagnosis of OC.

## 1. Introduction

Ovarian cancer (OC) is the most fatal gynecological malignancy worldwide with 286,000 occurrences and 176,000 deaths in 2017 [1]. Owing to the lack of specific symptoms, patients with OC often present with an advanced stage. Epithelial ovarian cancer is considered as the most common type of OC, with various subtypes of serous ovarian cancer (SOC), clear cell ovarian cancer, mucinous ovarian cancer, and endometrioid ovarian cancer (EOC) [2]. Surgery in combination with chemotherapy is the standard treatment recommended by the NCCN guideline for patients with OC of the advanced stage. Nevertheless, 5-year survival is only about 30% to 40% in most countries [3]. Therefore, reliable prognostic biomarkers for OC are needed to improve clinical outcomes.

Insulin-like growth factor binding proteins (IGFBPs), a family of secreted proteins that originally characterized as passive carriers of insulin-like growth factors (IGFs) in the circulation with high-affinity, are composed of 6 identified members (IGFBP1, 2, 3, 4, 5, 6) [4]. Apart from functions within the IGF system, they are acknowledged to play various roles in the extracellular and intracellular circumstances to modulate cell proliferation and apoptosis, as well as survival [5].

The relationships between IGFBPs and cancer prognosis remain contradictory in many studies. IGFBPs may increase cell survival and promote proliferation, while under other conditions, they may suppress tumor growth by stimulating apoptosis and suppressing cell proliferation [4]. Furthermore, there are no consistent or definitive evidences regarding the prognostic impact of IGFBPs in OC. In our study, we assessed the mRNA levels and protein levels of the IGFBP family in OC tissues and normal samples for the first time by using Oncomine datasets, Gene Expression Profiling Interactive Analysis (GEPIA) database, and the Human Protein Atlas (HPA) database. Moreover, we explored the prognosis of the six IGFBP genes in patients with OC through Kaplan-Meier plotter (KM plotter).

## 2. Materials and Methods

### 2.1. Oncomine Analysis

The Oncomine database [6] (https://www.oncomine.org), an internet cancer microarray platform containing 715 datasets and 86733 samples, was used to evaluate the IGFBP mRNA expression among various types of carcinoma with cut-off defined as *p* value =0.01, fold change (FC) “2”, and gene rank top 10%. Furthermore, we assessed the mRNA expression of individual IGFBPs between OC and healthy samples by Student's *t*-test. We set up cut-off at *p* value =0.01, fold change “2”, and gene rank top 10% as well.

### 2.2. GEPIA

GEPIA (http://gepia.cancer-pku.cn) is a network-based tool for processing the RNA expression information, collected from 9,736 carcinomas and 8,587 healthy specimens from GTEx project and TCGA. GEPIA offers diversified functions covering differential expression analysis, profile plotting, survival analysis, and correlation analysis [7]. We explored the IGFBP mRNA expression between SOC and healthy samples, which was evaluated by Student's *t*-test. The IGFBP mRNA expression among different pathological stages of SOC was assessed by the *F*-test. Foldchange > 2 and *p* < 0.01 were considered significant.

### 2.3. HPA

HPA (https://www.proteinatlas.org) is a valuable database providing human transcriptomic and proteomic information presented through Tissue Atlas, Pathology Atlas, and Cell Atlas among 44 various healthy organs, tissues, and 20 kinds of neoplasm [8]. It maps protein levels in normal samples and carcinoma tissues by utilizing immunohistochemistry. In our study, we used HPA to reveal the protein levels of the IGFBP family members in healthy specimens, SOC, and EOC tissues to evaluate whether it is in accordance with the mRNA expression from the GEPIA database.

### 2.4. KM Plotter

KM plotter [9] (http://kmplot.com/analysis/) was applied to analyze overall survival (OS) and progression-free survival (PFS) of the individual IGFBP mRNA level across 2190 OC patients. Specifically, clinical characteristics consisting of pathological histology, pathological grade, clinical stage, and chemotherapeutic regimen were collected from this database. The patient specimens were separated into “low” and “high” expression groups based on the mRNA expression of IGFBPs with established threshold values. The hazard ratio (HR), 95% confidence intervals (CIs), and *p* value were evaluated. Briefly, IGFBP1-6 were calculated in the datasets, respectively, to obtain the Kaplan-Meier survival plots. We defined *p* < 0.05 as statistically significant.

## 3. Results

### 3.1. IGFBP mRNA Expression in OC Patients

**Figure 1:**
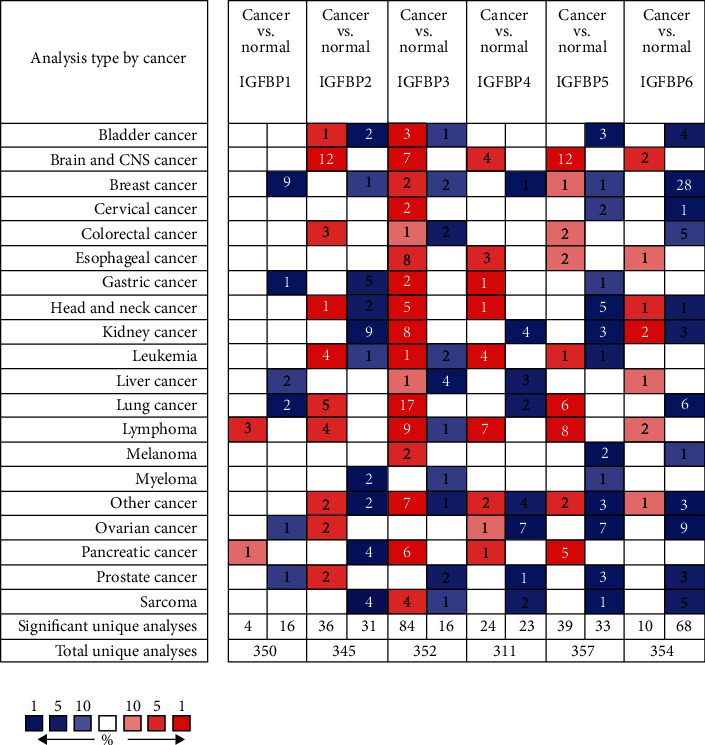
The mRNA levels of six IGFBPs in various kinds of carcinomas using the Oncomine database. The graph demonstrates the numbers of datasets with remarkably upregulated (red) and downregulated mRNA levels (blue) of the target subtype. The cut-off of *p* value and fold change was defined as 0.01 and 2, respectively.

**Figure 2:**
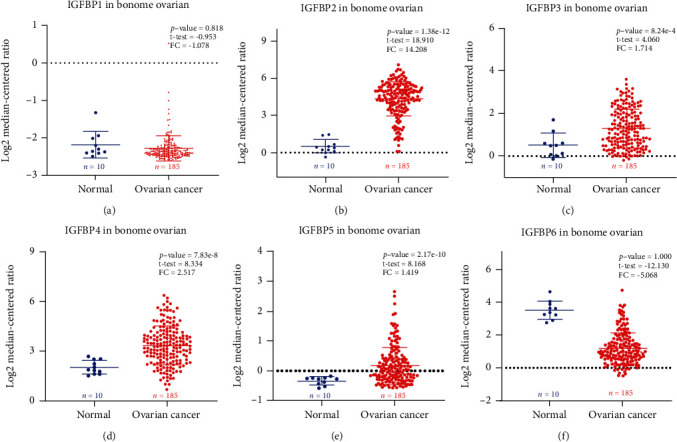
The mRNA levels of six IGFBPs in ovarian cancer and normal ovarian samples using the Oncomine database. (a) IGFBP1. (b) IGFBP2. (c) IGFBP3. (d) IGFBP4. (e) IGFBP5. (f) IGFBP6. The *p* value was defined as 0.01, while fold change was set up at 2.

With the Oncomine database, we analyzed the mRNA levels of the IGFBP family in various kinds of carcinomas compared with healthy specimens (Figure 1). Subsequently, mRNA levels of individual IGFBPs between OC and normal samples were evaluated as demonstrated in Figure 2. In the study of Bonome Ovarian, we observed that IGFBP2 and IGFBP4 were significantly upregulated in OC compared to healthy specimens [10].

**Figure 3:**
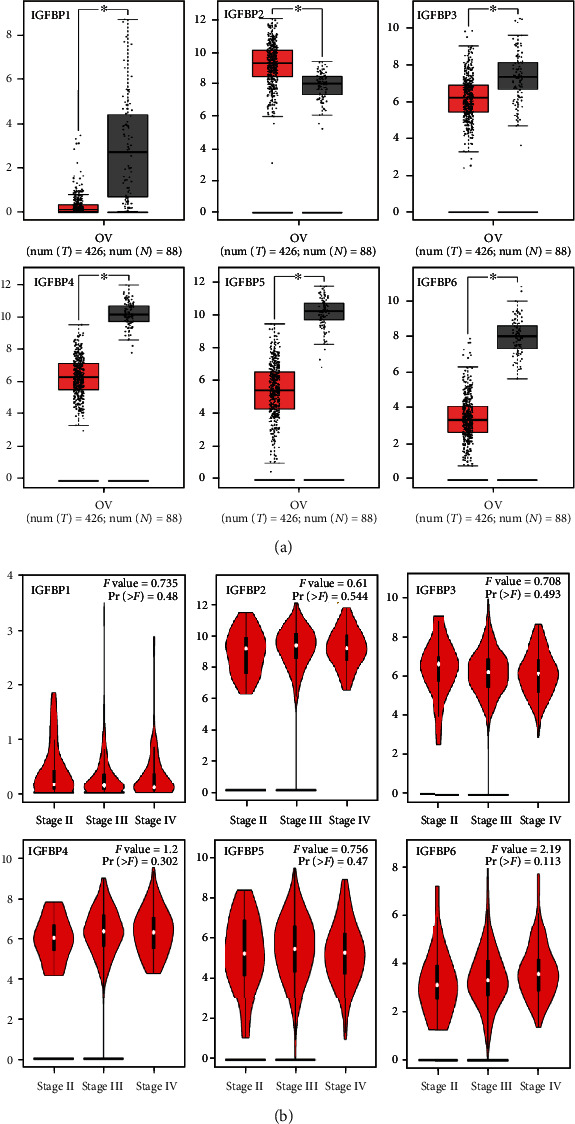
(a) The mRNA expression of six IGFBPs in women with serous ovarian cancer. (b) The levels of IGFBPs in the pathological stage II, III, and IV of serous ovarian cancer (GEPIA database). The cut-off of *p* value and fold change was set up at 0.01 and 2, respectively.

With the GEPIA database, we evaluated the mRNA expression of IGFBP genes in SOC samples and normal specimens. IGFBP1, IGFBP3, IGFBP4, IGFBP5, and IGFBP6 mRNA levels were remarkably downregulated in SOC than in healthy ovarian tissues, while IGFBP2 was remarkably higher (Figure 3(a)). No significant difference was found in mRNA levels of IGFBPs in different tumor pathological stages of SOC (Figure 3(b)).

### 3.2. IGFBP Protein Expression in OC Patients

**Figure 4:**
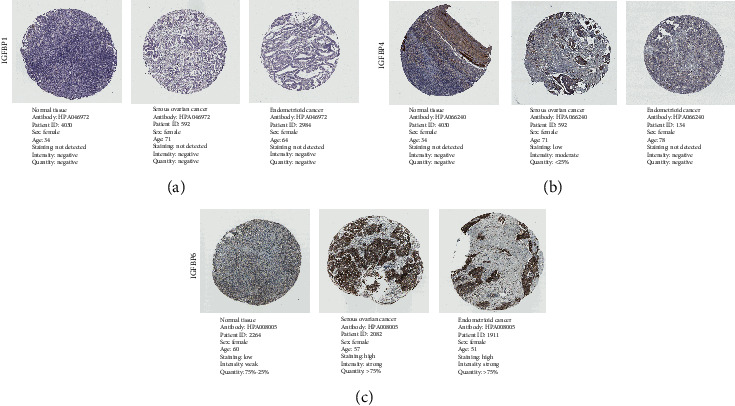
The protein expression of IGFBP1, IGFBP4, and IGFBP6 from the HPA database (The staining images of IGFBP2, IGFBP3, and IGFBP5 were unavailable). (a) IGFBP1. (b) IGFBP4. (c) IGFBP6.

With immunohistochemistry staining provided by the HPA, we assessed the IGFBP protein levels between healthy ovarian samples and OC tissues, including SOC and EOC tissues (Figure 4). The immunohistochemical staining images of IGFBP2, IGFBP3, and IGFBP5 are not provided in this study due to its unavailability in the HPA. Both of IGFBP1 and IGFBP4 showed no staining detected in normal ovarian tissues (Figures 4(a) and 4(b)). Using the exact same antibody applied to the 7 examined SOC, there were one case of low staining and six cases of not detected. In addition, all three examined EOC tissues revealed no detection of staining in both of IGFBP1 and IGFBP4. For IGFBP6, stroma cells demonstrated low staining in normal samples (Figure 4(c)). Among six detected SOC tissues, two cases with high staining of IGFBP6, two cases with medium staining, and two cases with low staining were observed. Among the two examined EOC samples, there were one case of high staining and one case of medium staining.

### 3.3. Prognostic Values of IGFBP mRNA Levels in OC Patients

**Figure 5:**
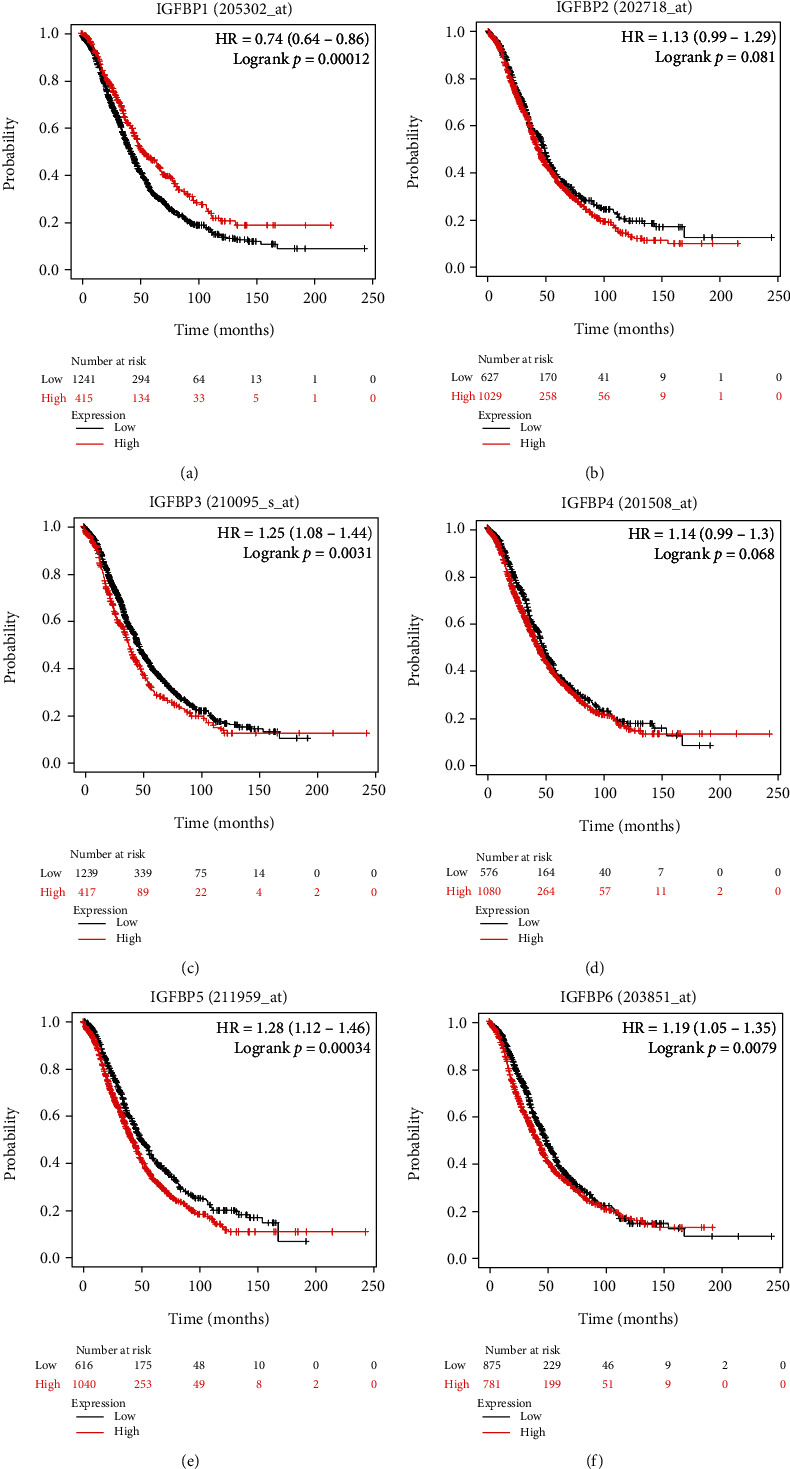
The prognostic impact of six IGFBPs regarding overall survival (OS) of all ovarian cancer using the Kaplan-Meier plotter. (a) The OS curves of IGFBP1 (*n* = 1656). (b) The curves of OS were plotted for IGFBP2 (*n* = 1656). (c) The OS curves of IGFBP3 with *n* = 1656. (d) The curves of OS were plotted for IGFBP4 (*n* = 1656). (e) The OS curves of IGFBP5 with *n* = 1656. (f) The OS curves of IGFBP6 (*n* = 1656). The curves of OS comparing patients with low (black) and high (red) IGFBP mRNA expression were plotted, with a cut-off *p* value of <0.05.

**Figure 6:**
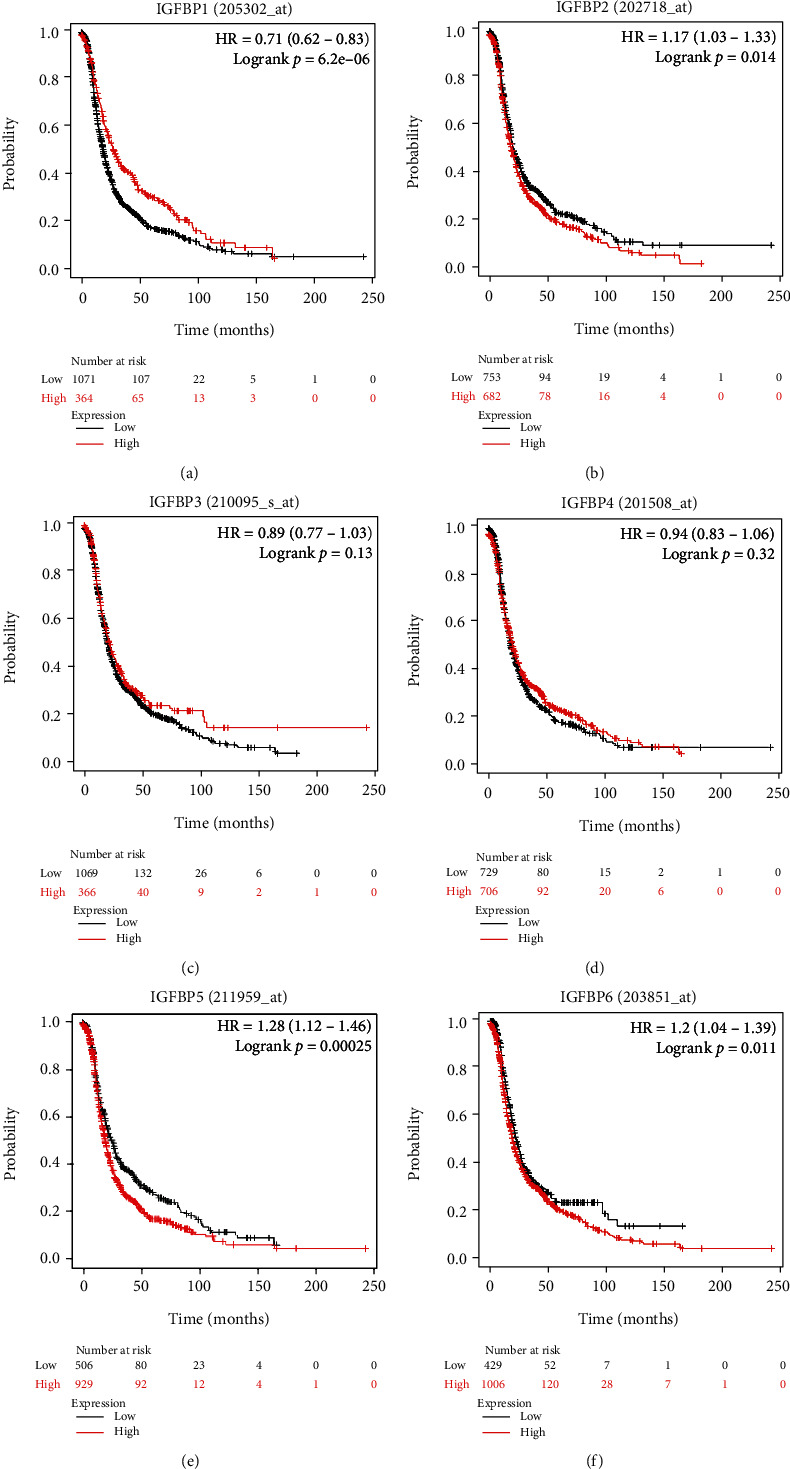
The prognostic value of the IGFBP family in progression-free survival (PFS) of all ovarian cancer using the Kaplan-Meier plotter. (a) The curves of PFS were plotted for IGFBP1 (*n* = 1435). (b) The PFS curves of IGFBP2 with *n* = 1435. (c) The curves of PFS were plotted for IGFBP3 with *n* = 1435. (d) Plotted PFS curves for IGFBP4 (*n* = 1435). (e) The curves of PFS were plotted for IGFBP5 (*n* = 1435). (f) Plotted PFS curves for IGFBP6 with *n* = 1435. The PFS curves comparing women with low (black) and high (red) IGFBP mRNA expression were plotted, with a cut-off *p* value of <0.05.

By using KM plotter, we further examined the prognostic impact of individual IGFBP mRNA levels among OC patients. Initially, we evaluated the prognostic significance of IGFBP1 (Figures 5(a) and 6(a) and Table 1). Elevated IGFBP1 mRNA levels were linked to better OS and PFS in all OC patients and a positive PFS in EOC patients. Furthermore, high expressed IGFBP1 mRNA was related to a better PFS in clinical stage I and II OC patients. Higher IGFBP1 was correlated with better OS and PFS in all OC patients receiving platin chemotherapy regimen.

Next, the prognostic effect of IGFBP2 was assessed (Figures 5(b) and 6(b) and Table 2). The overexpression of IGFBP2 mRNA indicated unfavorable PFS among all OC, EOC, and clinical stage I and II OC patients. Additionally, higher expression of IGFBP2 mRNA predicted a worsen OS in all OC patients received platin chemotherapeutic treatment.

Subsequently, the prognosis of the IGFBP3 was explored (Figures 5(c) and 6(c) and Table 3). Highly expressed IGFBP3 mRNA predicted worse OS in all OC, SOC, pathological grade II OC, and clinical stage III and IV OC, and all OC patients received platin chemotherapy regimen. However, IGFBP3 predicted positive PFS in EOC, and all OC patients received taxol and taxol+platin chemotherapeutic regimens.

As demonstrated in Figures 5(d) and 6(d) and Table 4, the overexpression of IGFBP4 was relevant to worsen OS and PFS in SOC and pathological grade II OC patients. High IGFBP4 was linked to worse PFS in pathological grade III OC and clinical stage III and IV OC patients. Furthermore, elevated IGFBP4 mRNA indicated a worsen OS in all OC patients that received platin chemotherapeutic regimen and worse PFS in all OC patients that received taxol and taxol+platin chemotherapy, whereas IGFBP4 revealed better PFS in 51 patients with EOC, 37 patients with pathological grade I OC, and 163 patients with clinical stage I and II OC.

We then assessed the prognostic impact of the IGFBP5 mRNA expression (Figures 5(e) and 6(e),and Table 5). The overexpression of IGFBP5 mRNA was in relation to worsen OS and PFS in all OC, pathological grade II OC patients, and clinical stage I and II OC patients. IGFBP5 predicted poor OS in SOC and EOC patients as well. However, IGFBP5 demonstrated a positive PFS in women with pathological grade III OC. Additionally, the overexpression of IGFBP5 predicted worse OS in all OC women received platin, taxol and taxol+platin chemotherapy, and unfavorable PFS in all patients treated with plain and taxol+platin chemotherapeutic regimens.

The prognosis of IGFBP6 was further detected (Figures 5(f) and 6(f) and Table 6). High expressed IGFBP6 mRNA was linked to worsen OS and PFS in all OC, SOC, and pathological grade III OC patients. IGFBP6 also showed worse PFS in pathological grade II OC and clinical stage III and IV OC patients. Further studies presented that high IGFBP6 was in relation to a poor OS in all OC patients received with platin chemotherapy and poor PFS in all OC women receiving taxol and taxol+platin chemotherapeutic treatments.

## 4. Discussion

IGFBP family plays a crucial role in modulating essential biological activities in the extracellular and intracellular compartments, such as cell proliferation, angiogenesis, apoptosis, survival, migration, and differentiation [4, 11]. Under various physiological circumstances, IGFBPs can mediate cellular functions through IGF-dependent or IGF-independent pathways. To our knowledge, we first used bioinformatic methods to evaluate the impact of the IGFBP family comprehensively on OC.

IGFBP1 has increased cell proliferation, cell-matrix adhesion, and regulate survival in schwannoma cells through the integrin *β*1/Src/FAK pathway [12]. IGFBP1 has also acted as a tumor inhibitor in hepatocellular cancer through downregulation of the MMP expression [13]. Meanwhile, findings on the correlation between the IGFBP1 and prognostic value in different types of carcinoma were inconsistent. Previous researches have revealed that the elevated expression of IGFBP1 is related to shorter time of metastasis and lower survival in gastric carcinoma [14] and prostate cancer [15]. However, several studies have revealed that low levels of IGFBP1 are related to increased risk of tumor progression and significant poor survival in breast cancer [16], colorectal cancer [17], and hepatocellular carcinoma [13]. Studies on the relationship between the predictive values of IGFBP1 and OC are limited. In ovarian clear cell adenocarcinoma, IGFBP1 has specifically expressed in both protein level and mRNA level by using immunohistochemistry and in situ hybridization [18]. A case-control study demonstrated that higher levels of IGFBP1 in plasma were correlated with higher risk of OC [19]. Our results with GEPIA datasets demonstrated that IGFBP1 mRNA levels were significantly downregulated in SOC. Furthermore, KM plotter revealed for the first time that highly expressed IGFBP1 mRNA indicated favorable OS and PFS in all OC and better PFS in women with EOC and clinical stage I and II OC. IGFBP1 also predicted favorable OS and PFS in all OC patients received with platin chemotherapy regimen. Evidence has been accumulated together that elevated levels of IGFBP1 may serve as a significant favorable prognostic predictor in all OC patients, particularly among patients with early stage and patients who received platin chemotherapy.

IGFBP2 ranks the second most abundant IGFBP in the circulation [20]. Some publications have suggested that IGFBP2 plays a tumor promotor role in glioblastoma through activation of *β*-catenin or EGFR–STAT3 pathways [21, 22], pancreatic ductal adenocarcinoma via activation of the NF-*κ*B pathway [23], and prostate cancer cells by the androgen-mediated pathway and MAPK-PI3K pathway [24]. Kang et al. have believed that the attenuated expression of IGFBP2 is in relation to worsen OS in rhabdomyosarcoma [25]. However, various studies have clarified that the overexpression of IGFBP2 are related to poorer prognosis in breast cancer [26], endometrial cancer [27], colorectal cancer [28], pancreatic ductal adenocarcinoma [23], lung cancer [29], and glioblastoma [21]. Despite of these controversial results, researches on OC seemed to be consistent. It has been previously reported that increased serum levels of IGFBP2 are linked with tumor grades and stages, predicting a higher recurrent risk and a shortened OS in OC [30]. Similarly, augmented IGFBP2 serum levels are correlated with poorer clinicopathological features and worse prognosis in epithelial ovarian cancer [31]. Besides, Huang et al. have also reported that IGFBP2 levels are higher in patients with worse responses to chemotherapy, which means IGFBP2 might act as a predictor of chemotherapy responses, especially in older patients or patients with SOC. In the present study, Oncomine and GEPIA analyses indicated that IGFBP2 mRNA levels were remarkably upregulated in both of OC and SOC in comparison with healthy tissues. Besides, our data from KM plotter datasets found that highly expressed IGFBP2 levels were link to a worse PFS in all OC patients and EOC patients. Furthermore, IGFBP2 also predicted a worsen OS in all OC patients received with platin treatment. Taken together, IGFBP2 may predict unfavorable clinical outcomes in women with OC.

IGFBP3, the most abundant circulating IGFBP, transports around 75% IGFs in heterotrimeric complexes including acid-labile subunit [5]. IGFBP3 exhibits antitumor effect in prostate carcinoma through crosstalk with the NF-*κ*B pathway and activation of caspase-dependent apoptosis [32]. Moreover, the overexpression of IGFBP3 is correlated with better respond rate to first-line chemotherapy and extended time of tumor progression and OS among metastatic colorectal cancer patients without receiving treatment previously [33]. Numerous researches have pointed out that the IGFBP3 overexpression is also in relation with the favorable survival in lung cancer [34], hepatocellular carcinoma [35], breast cancer [36], bladder carcinoma [37], esophageal squamous cell cancer [38], and pancreatic ductal adenocarcinoma [39]. However, raised levels of IGFBP3 are correlated with reduced survival in glioblastoma multiforme [40]. Furthermore, IGFBP3 regulated by miR-19a-3p can inhibit the proliferation, migration, and invasion in OC cells [41]. Increased levels of IGFBP3 are closely associated with an early clinical stage, nonserous histology, optimal cytoreduction, and favorable OS and PFS in epithelial ovarian cancer [31]. In a study of EOC, IGFBP3 acted as an invasion-metastasis inhibitor through the IGF-independent pathway; besides, lower expression of IGFBP3 induced higher pathological grade, advanced clinical stage, and worse survival [42]. Consistently, our results also showed that IGFBP3 predicted favorable PFS in EOC patients. Nevertheless, we observed that the overexpression of IGFBP3 mRNA was related to worsen OS in all OC and SOC patients. Therefore, these findings suggest that further research on the role of IGFBP3 in OC is required.

Elevated expression of IGFBP4 can lead to positive disease-free survival and OS in breast carcinoma [43], while IGFBP4 has a tumor-promoting effect on renal cell carcinoma by activating the Wnt/*β*-catenin signaling pathway [44]. High expression of IGFBP4 is related to metastasis and worse median survival rate in lung cancer [45]. Additionally, Mosig et al. first observed that both of IGFBP4 serum and tumor levels were elevated among all stages of epithelial ovarian cancer patients [46]. Our results from the Oncomine database clarified that the IGFBP4 mRNA expression was significantly highly expressed in OC. Nevertheless, there is paucity of study regarding prognosis effects of IGFBP4 in OC. In this study, the overexpression of IGFBP4 was in relation to worsen OS and PFS in SOC patients. High IGFBP4 was linked to a worsen OS in all OC patients received with platin chemotherapy. Overall, these findings collectively suggested that IGFBP4 might predict a poor outcome in women with SOC.

IGFBP5 plays as a tumor suppressor in breast cancer through estradiol-triggered activation of the Akt/PKB pathway [47]. Lower serum levels of IGFBP5 are correlated with a positive lymph node status and poor recurrence-free survival in lung cancer [48]. However, the overexpression of IGFBP5 is related to poor prognosis in ER-negative breast cancer patients with positive lymph nodes [49]. Furthermore, elevated levels of IGFBP5 are correlated to advanced stages, worse disease-specific survival, and metastatic-free survival in urothelial cancers of upper urinary tracts and urinary bladder [50]. C-terminus of IGFBP5 exhibited antitumor effect by suppressing angiogenesis through the Akt/ERK and NF-*κ*B–VEGF/MMP-9 pathway in OC [51]. On the contrary, Wang et al. have detected increased IGFBP5 protein levels in high-grade SOCs in comparison with healthy surface epithelium, serous benign cysts, borderline serous neoplasms, and low-grade SOCs [52]. Discordant with this research, our data from the GEPIA database pointed out that IGFBP5 mRNA levels were remarkably reduced in SOC than in healthy specimens. Additionally, this present study is the first one to report on the prognosis of IGFBP5, which exhibited that high IGFBP5 was linked to poor OS and PFS in all OC patients and all OC patients that received with platin and taxol+platin treatments. IGFBP5 also predicted a worsen OS in all OC patients that received with taxol chemotherapeutic regimen. As pointed out above, IGFBP5 may predict a reduced prognosis in OC, especially in SOC patients. The overexpression of IGFBP5 may result in chemotherapy resistance in patients receiving platin and taxol+platin regimens.

IGFBP6 encourages migration in rhabdomyosarcoma cells via mediating the MAP kinase signaling pathway [53], while IGFBP6 plays a suppressive role on tumor growth in ACTH-secreting pituitary adenoma through activation of the PI3K-AKT-mTOR signaling pathway [54]. In addition, IGFBP6 is inversely associated with glioma grade and predicts better survival [55]. The low expression of IGFBP6 is related to poor clinical outcomes and unfavorable prognosis in gastric adenocarcinoma [56]. Moreover, IGFBP6 promotes migration in SKOV3 OC cells via activation of the MAP kinase signaling pathway, whereas IGFBP6 represses migration in HEY OC cells through both the IGF-dependent and IGF-independent pathway [57]. Gunawardana et al. noticed that the IGFBP6 expression in serum was significantly decreased in epithelial ovarian cancer compared to the healthy ovarian tissue [58]. Using information from HPA datasets, we observed that IGFBP6 protein levels were upregulated in both of SOC and EOC tissues when compared to normal tissues. However, there are limited studies on the prognosis of IGFBP6 in women with OC. Our data from the KM plotter database demonstrated that the overexpression of IGFBP6 was linked to worsen OS and PFS in all OC patients, SOC patients, and pathological grade III patients. Meanwhile, IGFBP6 predicted a worse OS in all OC patients received with platin treatment and worse PFS in pathological grade II patients, clinical stage III and IV patients, and all women received taxol and taxol+platin chemotherapeutic regimens. Accumulated evidence indicated that IGFBP6 may be a poor outcome predictor in OC, especially among poor differentiated and SOC patients.

## 5. Conclusions

Taken together, this study indicates that IGFBP2 and IGFBP4 are potential biomarkers for the diagnosis of OC. High IGFBP1 mRNA is in relation to positive OS and PFS in all OC patients. By contrast, high IGFBP5 and IGFBP6 mRNA levels are linked to worsen OS and PFS in all OC patients. Moreover, high IGFBP4 and IGFBP6 mRNA predict worsen OS and PFS in women with SOC. Different IGFBPs are associated with various pathological grades and clinical stages, and IGFBP1/2/4/5/6 are useful prognostic indicators for chemotherapeutic effect in patients with OC.

## Figures and Tables

**Table 1:** The prognostic value of the IGFBP1 mRNA expression in ovarian cancer.

	Overall survival			Progression-free survival		
	Cases	HR (95% CI)	*p* value	Cases	HR (95% CI)	*p* value
Histology						
All cancer patients	1656	0.74 (0.64−0.86)	0.00012^∗^	1435	0.71 (0.62−0.83)	6.2e−06^∗^
Serous cancer patients	1207	0.86 (0.72−1.03)	0.099	1104	0.91 (0.77−1.07)	0.24
Endometrioid cancer patients	37	2.66 (0.3−23.76)	0.36	51	0.21 (0.05−0.91)	0.021^∗^
Pathological grades						
I	56	0.44 (0.17−1.16)	0.09	37	0.33 (0.09−1.23)	0.084
II	324	0.76 (0.56−1.03)	0.08	256	0.87 (0.62−1.22)	0.42
III	1015	0.89 (0.74−1.08)	0.24	837	0.88 (0.73−1.07)	0.19
Clinical stages						
I and II	135	0.42 (0.17−1.04)	0.052	163	0.41 (0.19−0.87)	0.016^∗^
III and IV	1220	0.86 (0.74−1.01)	0.063	1081	1.11 (0.96−1.29)	0.15
Chemotherapy						
Contains platin	1409	0.79 (0.67−0.93)	0.005^∗^	1259	0.79 (0.69−0.91)	0.00094^∗^
Contains taxol	793	0.85 (0.68−1.05)	0.13	715	1.16 (0.96−1.41)	0.13
Contains taxol+platin	776	0.82 (0.65−1.02)	0.077	698	1.16 (0.95−1.41)	0.15

^∗^ *p* < 0.05.

**Table 2:** The prognostic value of the IGFBP2 mRNA expression in ovarian cancer.

	Overall survival			Progression-free survival		
	Cases	HR (95% CI)	*p* value	Cases	HR (95% CI)	*p* value
Histology						
All cancer patients	1656	1.13 (0.99−1.29)	0.081	1435	1.17 (1.03−1.33)	0.014^∗^
Serous cancer patients	1207	0.87 (0.74−1.02)	0.079	1104	1.13 (0.98−1.3)	0.098
Endometrioid cancer patients	37	4.93 (0.55−44.16)	0.11	51	2.67 (1.05− 6.79)	0.032^∗^
Pathological grades						
I	56	0.55 (0.22−1.42)	0.21	37	1.76 (0.58−5.41)	0.31
II	324	1.31 (0.95−1.8)	0.096	256	1.21 (0.9−1.63)	0.2
III	1015	0.86 (0.73−1.02)	0.088	837	1.07 (0.91−1.27)	0.41
Clinical stages						
I and II	135	1.68 (0.77−3.67)	0.19	163	2.16 (1.1−4.25)	0.022^∗^
III and IV	1220	1.11 (0.95−1.28)	0.18	1081	0.91 (0.78−1.07)	0.27
Chemotherapy						
Contains platin	1409	1.2 (1.03−1.39)	0.02^∗^	1259	1.12 (0.98−1.27)	0.087
Contains taxol	793	1.16 (0.95−1.41)	0.16	715	0.91 (0.75−1.11)	0.35
Contains taxol+platin	776	1.15 (0.94−1.41)	0.17	698	1.08 (0.91−1.29)	0.38

^∗^ *p* < 0.05.

**Table 3:** The prognostic value of the IGFBP3 mRNA expression in ovarian cancer.

	Overall survival			Progression-free survival		
	Cases	HR (95% CI)	*p* value	Cases	HR (95% CI)	*p* value
Histology						
All cancer patients	1656	1.25 (1.08−1.44)	0.0031^∗^	1435	0.89 (0.77−1.03)	0.13
Serous cancer patients	1207	1.25 (1.05−1.48)	0.0099^∗^	1104	0.88 (0.75−1.05)	0.15
Endometrioid cancer patients	37	5.91 (0.66−52.98)	0.071	51	0.34 (0.12−0.95)	0.031^∗^
Pathological grades						
I	56	0.59 (0.23−1.53)	0.28	37	2.67 (0.89−7.96)	0.068
II	324	1.36 (1.01−1.84)	0.045^∗^	256	0.81 (0.58−1.13)	0.22
III	1015	0.88 (0.74−1.03)	0.12	837	0.83 (0.68−1.01)	0.062
Clinical stages						
I and II	135	1.56 (0.72−3.4)	0.26	163	0.59 (0.31−1.14)	0.12
III and IV	1220	1.2 (1.02−1.41)	0.028^∗^	1081	0.89 (0.75−1.05)	0.15
Chemotherapy						
Contains platin	1409	1.21 (1.04−1.41)	0.014^∗^	1259	1.12 (0.99−1.28)	0.078
Contains taxol	793	1.15 (0.94−1.4)	0.17	715	0.82 (0.67−1)	0.049^∗^
Contains taxol+platin	776	1.13 (0.92−1.38)	0.24	698	0.8 (0.65−0.98)	0.029^∗^

^∗^ *p* < 0.05.

**Table 4:** The prognostic value of the IGFBP4 mRNA expression in ovarian cancer.

	Overall survival			Progression-free survival		
	Cases	HR (95% CI)	*p* value	Cases	HR (95% CI)	*p* value
Histology						
All cancer patients	1656	1.14 (0.99−1.3)	0.068	1435	0.94 (0.83−1.06)	0.32
Serous cancer patients	1207	1.17 (1−1.37)	0.047^∗^	1104	1.24 (1.07−1.44)	0.005^∗^
Endometrioid cancer patients	37	2.81 (0.31−25.11)	0.34	51	0.1 (0.01−0.79)	0.0077^∗^
Pathological grades						
I	56	0.19 (0.03−1.47)	0.076	37	0.24 (0.05−1.09)	0.045^∗^
II	324	1.41 (1.04−1.92)	0.026^∗^	256	1.52 (1.11−2.09)	0.0085^∗^
III	1015	1.18 (0.98−1.42)	0.08	837	1.23 (1−1.5)	0.045^∗^
Clinical stages						
I and II	135	1.47 (0.67−3.21)	0.33	163	0.34 (0.19−0.6)	8.7e−05^∗^
III and IV	1220	1.16 (0.99−1.35)	0.065	1081	1.34 (1.15−1.56)	0.00015^∗^
Chemotherapy						
Contains platin	1409	1.24 (1.06−1.44)	0.0067^∗^	1259	1.1 (0.96−1.27)	0.17
Contains taxol	793	1.15 (0.95−1.4)	0.15	715	1.21 (1.02−1.45)	0.029^∗^
Contains taxol+platin	776	1.18 (0.97−1.43)	0.1	698	1.23 (1.03−1.47)	0.022^∗^

^∗^ *p* < 0.05.

**Table 5:** The prognostic value of the IGFBP5 mRNA expression in ovarian cancer.

	Overall survival			Progression-free survival		
	Cases	HR (95% CI)	*p* value	Cases	HR (95% CI)	*p* value
Histology						
All cancer patients	1656	1.28 (1.12−1.46)	0.00034^∗^	1435	1.28 (1.12−1.46)	0.00025^∗^
Serous cancer patients	1207	1.19 (1.01−1.4)	0.043^∗^	1104	0.92 (0.8−1.06)	0.25
Endometrioid cancer patients	37	6.8 (0.76−60.94)	0.047^∗^	51	1.95 (0.77−4.96)	0.15
Pathological grades						
I	56	1.43 (0.54−3.78)	0.47	37	2.67 (0.87−8.23)	0.075
II	324	1.5 (1.07−2.12)	0.018^∗^	256	1.57 (1.15−2.15)	0.004^∗^
III	1015	1.08 (0.91−1.29)	0.37	837	0.83 (0.7−0.98)	0.026^∗^
Clinical stages						
I and II	135	2.56 (1.07−6.14)	0.029^∗^	163	2.08 (1.15−3.77)	0.014^∗^
III and IV	1220	1.13 (0.96−1.32)	0.14	1081	0.9 (0.79−1.04)	0.16
Chemotherapy						
Contains platin	1409	1.27 (1.09−1.47)	0.0017^∗^	1259	1.27 (1.1−1.47)	0.00099^∗^
Contains taxol	793	1.37 (1.12−1.67)	0.002^∗^	715	1.2 (0.99−1.47)	0.063
Contains taxol+platin	776	1.4 (1.14−1.71)	0.0012^∗^	698	1.21 (1.01−1.46)	0.04^∗^

^∗^ *p* < 0.05.

**Table 6:** The prognostic value of the IGFBP6 mRNA expression in ovarian cancer.

	Overall survival			Progression-free survival		
	Cases	HR (95% CI)	*p* value	Cases	HR (95% CI)	*p* value
Histology						
All cancer patients	1656	1.19 (1.05−1.35)	0.0079^∗^	1435	1.2 (1.04−1.39)	0.011^∗^
Serous cancer patients	1207	1.27 (1.09−1.49)	0.0023^∗^	1104	1.29 (1.12−1.49)	0.00046^∗^
Endometrioid cancer patients	37	0.32 (0.05−1.92)	0.19	51	1.76 (0.58−5.35)	0.31
Pathological grades						
I	56	1.48 (0.55−3.96)	0.43	37	2.68 (0.9−7.98)	0.066
II	324	1.29 (0.95−1.74)	0.097	256	1.7 (1.22−2.39)	0.0017^∗^
III	1015	1.27 (1.06−1.53)	0.0084^∗^	837	1.31 (1.11−1.54)	0.0015^∗^
Clinical stages						
I and II	135	0.53 (0.24−1.18)	0.11	163	1.42 (0.8−2.53)	0.23
III and IV	1220	1.14 (0.98−1.33)	0.082	1081	1.26 (1.09−1.45)	0.0015^∗^
Chemotherapy						
Contains platin	1409	1.21 (1.05−1.39)	0.0069^∗^	1259	1.12 (0.96−1.3)	0.15
Contains taxol	793	1.17 (0.97−1.41)	0.1	715	1.27 (1.05−1.53)	0.012^∗^
Contains taxol+platin	776	1.13 (0.93−1.37)	0.21	698	1.26 (1.05−1.53)	0.015^∗^

^∗^ *p* < 0.05.

## Data Availability

The data used in this article to support the findings are included in the documentation.
